# Early exposure to ultraviolet-B radiation decreases immune function later in life

**DOI:** 10.1093/conphys/cow037

**Published:** 2016-09-22

**Authors:** Emma Ceccato, Rebecca L. Cramp, Frank Seebacher, Craig E. Franklin

**Affiliations:** 1 School of Biological Sciences, University of Queensland, Brisbane, QLD 4072, Australia; 2 School of Life and Environmental Sciences, University of Sydney, Sydney, NSW 2006, Australia

**Keywords:** Amphibian declines, disease, immunocompetence, leucocyte, phytohaemagglutinin, ultraviolet radiation

## Abstract

Amphibians have declined dramatically worldwide. Many of these declines are occurring in areas where no obvious anthropogenic stressors are present. It is proposed that in these areas, environmental factors such as elevated solar ultraviolet-B (UV-B) radiation could be responsible. Ultraviolet-B levels have increased in many parts of the world as a consequence of the anthropogenic destruction of the ozone layer. Amphibian tadpoles are particularly sensitive to the damaging effects of UV-B radiation, with exposure disrupting growth and fitness in many species. Given that UV-B can disrupt immune function in other animals, we tested the hypothesis that early UV-B exposure suppresses the immune responses of amphibian tadpoles and subsequent juvenile frogs. We exposed *Limnodynastes peronii* tadpoles to sublethal levels of UV-B radiation for 6 weeks after hatching, then examined indices of immune function in both the tadpoles and the subsequent metamorphs. There was no significant effect of UV-B on tadpole leucocyte counts or on their response to an acute antigen (phytohaemagglutinin) challenge. However, early UV-B exposure resulted in a significant reduction in both metamorph leucocyte abundance and their response to an acute phytohaemagglutinin challenge. These data demonstrate that early UV-B exposure can have carry-over effects on later life-history traits even if the applied stressor has no immediately discernible effect. These findings have important implications for our understanding of the effects of UV-B exposure on amphibian health and susceptibility to diseases such as chytridiomycosis.

## Abstract (precis)

With frog populations in decline, it is important to understand the impact of changing environmental stressors, such as increased solar UV-B radiation, on frog health. This study investigated the impact of elevated UV-B radiation on tadpole immune function, and the carry-over effects of early UV-B exposure on immune function, post-metamorphosis.

## Introduction

Amphibians have been experiencing dramatic declines worldwide, with populations from 42% of species known to be decreasing (Alford and Richards, 1999; Stuart *et al*., 2004; IUCN, 2016). Most of these declines have been attributed to anthropogenic factors; however, some declines are more enigmatic, as they occur in habitats that are free from any obvious anthropogenic pressures (Stuart *et al*., 2004). In these areas, it has been hypothesized that altered environmental factors, such as increased ultraviolet-B (UV-B) radiation, could be responsible (Collins and Storfer, 2003).

The destruction of the ozone layer in the upper atmosphere has led to an increase in ultraviolet (UV) radiation reaching the Earth's surface (Kerr and McElroy, 1993; Herman *et al*., 1996; McKenzie *et al*., 1999; Stuart *et al*., 2004). Exposure to solar radiation in the UV-B range (290–320 nm) can cause a wide range of sublethal effects on eggs and tadpoles, including reduced growth rates (Ankley *et al*., 2002), increased occurrence of developmental mortalities (Pahkala *et al*., 2002), decreased locomotor performance (Blaustein *et al*., 1994) and altered behaviours (Kats *et al*., 2000; Han *et al*., 2007). Exposure to UV-B radiation also synergistically enhances the negative effects of other stressors (Kiesecker and Blaustein, 1995; Kats *et al*., 2000; Pahkala *et al*., 2002; Hatch and Blaustein, 2003; van Uitregt *et al*., 2007; Alton *et al*., 2010; Mitchell *et al*., 2012). Sensitivity to UV-B radiation varies between species (Blaustein *et al*., 1994) and between populations, with populations at higher elevations considered to be at greater risk of UV-B-associated damage than populations at lower elevations, because they receive higher levels of solar UV-B radiation (Belden and Blaustein, 2002).

The exact mechanisms underlying the effect of UV-B radiation on amphibians are unknown, although it is hypothesized that the damage to DNA caused by UV-B exposure may be responsible. Ultraviolet-B exposure results in the synthesis of pyrimidine dimers between adjacent DNA nucleotides, which impedes the transcription of the affected gene, leading to cellular mutation or death (Jagger, 1985; Hearst, 1995; Friedberg *et al*., 2006). Amphibians, like other vertebrates, have evolved DNA repair mechanisms to reverse the damage caused by UV-B radiation through the activation of the photo-repair enzymes, the photolyases (Hearst, 1995). However, this repair is considered energetically costly (Sancar and Tang, 1993) and, therefore, it has been proposed that a trade-off may exist between costly repair and ‘regular’ functions, such as growth and performance (Jagger, 1985; Sancar and Tang, 1993; Hearst, 1995; Friedberg *et al*., 2006; Alton *et al*., 2012).

Ultraviolet-B exposure suppresses immune function in many vertebrate species, including fish (Jokinen *et al*., 2008), mice (Kripke, 1984), rats (Goettsch *et al*., 1994) and humans (Poon *et al*., 2005). The mechanisms for this immunosuppressive effect vary from local damage or killing of important antigen-presenting cells in the skin (Stingl *et al*., 1983) to stimulation of keratinocytes to release cytokines that induce systemic immune suppression (Rivas and Ullrich, 1992) or, indirectly, through an increase in concentrations of corticosteroids (cortisol or corticosterone), important stress hormones that also have an immunosuppressive function (Jokinen *et al*., 2000). Given that the immune function of fish and mammals can be impaired through exposure to elevated UV-B, negative effects of exposure on the amphibian immune system are likely.

In recent years, the emergence of novel diseases and pathogens has had a devastating effect on amphibian populations worldwide (Carey *et al*., 1999). One of the most influential of amphibian pathogens is the fungus *Batrachochytrium dendrobatidis* (hereafter referred to as *Bd*), which causes the disease chytridiomycosis (Berger *et al*., 1998; Pessier *et al*., 1999). *Batrachochytrium dendrobatidis* has been documented in >500 amphibian species worldwide and is implicated in the decline or extinction of several hundred of these (van Rooij *et al*., 2015). Indeed, *Bd* is now thought to be responsible for the greatest loss of vertebrate biodiversity ever attributed to disease (Skerratt *et al*., 2007). Although recent data suggest that several *Batrachochytrium* lineages appear to have coexisted with amphibian populations for some time (James *et al*., 2015), it is still unclear whether the global devastation caused by *Bd* is solely the result of exposure to a novel lineage or whether exposure to environmental stressors, such as exposure to increases in UV-B radiation, may have increased the susceptibility of amphibians to this pathogen. Levels of UV-B radiation have increased substantially over recent decades in many regions of the world (Herman, 2010). Ultraviolet-B suppresses immune function and impairs resistance to pathogens in fish and mammals (Salo *et al*., 2000; Jokinen *et al*., 2008), but its effects on amphibian immune function remain largely untested.

Although the direct effects of elevated UV-B exposure on eggs and tadpoles are well established, the potential for these effects to persist into other life-history stages, known as carry-over effects, remains unexplored. Environmental stress during embryonic and larval stages in amphibians can impact on locomotion (Boes and Benard, 2013), morphology (Relyea, 2001; Tejedo *et al*., 2010) and survival in later life-history stages (Rasanen *et al*., 2002). Exposure of embryos to UV-B has been shown to elicit carry-over effects in the form of increased occurrence of developmental abnormalities and slower development in the subsequent *Rana temporaria* tadpoles, despite having no apparent effect on the embryonic stage (Pahkala *et al*., 2001). Therefore, it is possible that the effect of exposure to elevated UV-B in early life-history stages on immune function in tadpoles could persist and impact upon fitness in later life.

To investigate the impact of early (larval) UV-B exposure on tadpole and subsequent metamorph immune function, we exposed tadpoles of the brown striped marsh frog, *Limnodynastes peronii*, to sublethal ecologically relevant levels of UV-B radiation for 6 weeks and measured several indices of physiological performance and immune function in the tadpoles and resulting metamorphs. The tadpoles of *L. peronii* are sensitive to the ecologically relevant levels of UV-B used in the present study (van Uitregt *et al*., 2007; Alton *et al*., 2011; Bernal *et al*., 2011). *Limnodynastes peronii* produces foamy egg masses, which are laid on the water surface during spring and summer in both open and shaded positions (Anstis, 2002). Larvae then hatch from the foam nest after a few days and remain free swimming in the water until metamorphosis. Given that early life-history exposure to other environmental stressors can have a lasting effect on post-metamorphic traits, we hypothesized that the sublethal exposure of early tadpoles to UV-B would disrupt immune function in both the tadpoles and the subsequent metamorphs.

## Materials and methods

### Ethics statement

This research was approved by the University of Queensland Animal Welfare Unit (permit number SBS/085/13/URG) and the Queensland Department of Environment and Heritage (WISP07785810).

### Animal collection and maintenance

Four recently laid *L. peronii* egg foam masses were collected from The University of Queensland campus, Brisbane, Australia (27°29′′50.54″S, 153° 1′4.12″E). Seven days after hatching, 210 tadpoles were distributed evenly among 21 2 litre containers, each containing 1 litre of aged tap water. Twelve containers were randomly assigned to a UV-B treatment (*n* = 120 individual tadpoles) and nine to the control treatment (*n* = 90). The larger sample size of tadpoles assigned to the UV-B treatment was to account for any mortality expected to be experienced in the UV-B treatment group. Tadpoles were kept at 24 ± 2°C and were fed frozen spinach twice a week. Ambient, (non-UV) fluorescent lighting in the room maintained the photoperiod at 12 h light–12 h dark.

### Ultraviolet-B treatments

Ultraviolet radiation was emitted from four fluorescent sources [(Reptile One T8 36 W) two UV 5.0 and two UV 10.0]. Control group containers were protected from UV-B by placement of metallized window film (Clear grey; Handi Home Supplies, Thomastown, Victoria, Australia), which was suspended over the control containers. The lights were set to a regimen in which the UV lights would emit a baseline level of UV-B from 10.00 to 11.00 h and again from 13.00 to 14.00 h daily, with a peak level of UV-B delivered during the daily midpoint (10.00–13.00 h; Table 1). After 20 days (beginning during week 3 of the exposure period and continuing until the completion of the exposure period in week 6), levels of UV-B were increased by replacement with stronger UV-B lights [two UV-B 8.0 (Repti-Glo Exo-Terra 40 W) and two UV 10.0 (Reptile One T8 36 W)] that emitted UV-B radiation simultaneously for 4 h daily (10.00–14.00 h; Table 2).

**Table 1:** Calculated UV spectra measurements, including absolute irradiance levels of UV-A and UV-B and absolute cumulative daily does rates of baseline and peak levels of UV-B radiation during the initial exposure period (experienced during weeks 0–3 of the exposure period)

Fluctuating daily cycle	Baseline irradiance (µW cm^−2^), 4 h day^−1^		Peak irradiance (µW cm^−2^), 2 h day^−1^		Cumulative daily dose (kJ m^−2^)	
	UV-B	UV-A	UV-B	UV-A	UV-B	UV-A
UV-B treatment	7.59 ± 1.59	83.85 ± 16.04	12.90 ± 3.18	63.89 ± 10.87	1.904 ± 0.394	11.468 ± 2.062
Control treatment	1.42 ± 0.95	3.05 ± 0.70	−2.08 ± 1.93	1.62 ± 0.99	0.076 ± 0.199	0.521 ± 0.167

Values are means ± SD. Abbreviation: UV, ultraviolet.

**Table 2:** Calculated UV spectra measurements, including absolute irradiance levels of UV-A and UV-B and absolute cumulative daily does rates of baseline levels of UV-B radiation during the final exposure period (experienced during weeks 3–6 of the exposure period)

Daily cycle	Absolute irradiance (µW cm^−2^), 4 h day^−1^		Cumulative daily dose (kJ m^−2^)	
	UV-B	UV-A	UV-B	UV-A
UV-B treatment	18.41 ± 3.55	132.48 ± 21.67	2.43 ± 0.65	19.06 ± 3.25
Control treatment	1.65 ± 0.64	9.80 ± 1.90	0.24 ± 0.10	1.40 ± 0.29

Values are means ± SD. Abbreviation: UV, ultraviolet.

This increase in UV-B radiation of was applied to account for the increased water depth of the containers as the tadpoles progressed through development.

All UV-B treatment levels were substantially lower than the ambient levels of 500 µW cm^−2^ measured during summer in the middle of the day in Brisbane recorded by van Uitregt *et al*. (2007). Given that UV-B penetration into water is attenuated by suspended particulate matter and dissolved carbon, it is likely that tadpoles would experience only a small fraction of the total solar irradiance reported by van Uitregt *et al*. (2007). Ultraviolet-B radiation was measured using a cosine corrector (CC-3-UV-S; Ocean Optics, Dunedin, FL, USA) and fibre-optic cable (400 µm Premium Fiber; Ocean Optics) attached to a spectrometer (USB2000+ Miniature Fiber Optic Spectrometer; Ocean Optics). Measurements were taken from the midpoint position of each container at the level of the water surface. The irradiance of UV-B and ultraviolet-A (UV-A) at each container midpoint was calculated by integrating the spectral irradiance data between 300 and 320 nm and between 320 and 400 nm, respectively (Alton *et al*., 2011).

After the 40 day exposure period, the UV lights were switched off, and a subset of tadpoles was tested immediately for immune function [leucocyte count and response to phytohaemagglutinin (PHA)]. The remaining tadpoles were maintained under 12 h–12 h rooftop fluorescent lighting until metamorphosis (determined as the point at which the tail was fully resorbed). Metamorphs were measured for immunocompetence (leucocyte counts and response to PHA) 14 days after each metamorph had achieved metamorphosis. By measuring each metamorph 14 days after metamorphosis the age at which all the metamorphs underwent the analysis of immune function was kept constant. This was done to avoid testing in the immunosuppressed stage of development that follows metamorphosis prior to the development of the adult-type immune system (Rollins-Smith, 1998).

### Growth rate measurements

Growth rates were measured periodically during larval development (0, 2 and 4 weeks after UV-B exposure had begun) and upon completion of metamorphosis (i.e. tail reabsorption stage completed). Photographs were taken fortnightly to determine the growth rate at three different time points during the exposure period. Four tadpoles from each container were photographed with a digital camera (EX-ZR200 Exilim; Casio, Tokyo, Japan) for size measurements, such that 48 tadpoles from the UV-B treatment group and 36 tadpoles from control group were analysed at each time point. Total length measurements, the length from the top of the head to the end of the tail, of each tadpole were measured from the images using the analysis program ImageJ (National Institutes of Health, Bethesda, MD, USA). The size at metamorphosis was determined by analysing the mass and snout-vent length of the animals measured once metamorphosishad been completed (defined as larval tail resorbed to < 2 mm in length).

### Metabolic rate of tadpoles

To assess the metabolic cost of UV-B exposure for tadpoles, the standard metabolic rate was determined at two different time points during UV-B exposure; firstly, during the initial UV-B exposure period (experienced from the beginning of the exposure period to week 3; Gosner developmental stage 21–25 (Gosner, 1960); absolute irradiance of UV-B during peak = 7.59 ± 1.59 µW cm^−2^, mean ± SEM) and secondly, during the final UV-B exposure period (experienced from week 3 until the completion of exposure in week 10; Gosner developmental stage 25–38 (Gosner, 1960); absolute irradiance of UV-B during peak = 18.41 ± 3.55 µW cm^−2^, mean ± SEM). Measurements were taken from three tadpoles per container from UV-B and control groups (initial exposure period, *n* = 36 and *n* = 27, respectively; and final exposure period, *n* = 25 and *n* = 27, respectively). Metabolic rate was measured using closed-system respirometry, by placing each tadpole in a 25 ml syringe equipped with an integrated oxygen-sensitive fluorescent Sensor Spot (PreSens, Regensburg, Germany) filled with air-saturated aged water. The oxygen concentration was measured every 30 min for an hour using a Fibox3 reader (PreSens).

The oxygen consumption rate (${\dot{V}}_{O_{2}}$; in millilitres of O_2_ per hour) of the tadpoles was corrected for water temperature and for any background respiration, using the following formula:

$${\dot{V}}_{O_{2}}={(\Delta O}_{2}\times V)/t$$

where ΔO_2_ is the change in oxygen concentration after accounting for background respiration (in millilitres of O_2_ per litre), *V* is the volume of the syringe (in millilitres) and *t* is time (in minutes). Body mass was incorporated into statistical analysis as a covariate.

### Phytohaemagglutinin challenge

A subset of tadpoles from the UV-B treatment group (*n* = 10) and the control group (*n* = 7) were lightly anaesthetized in Aqualife TMS (Syndel Laboratories, Nanaimo, BC, Canada; 0.125 g l^−1^), and the dorsal plane of each tadpole was photographed. Tadpoles’ tails were then injected with 3 µl of PHA (12 mg ml^−1^; Sigma-Aldrich, St Louis, MO, USA) dissolved in sterile amphibian Ringer solution (6.6 g NaCl, 0.15 g KCl, 0.15 g CaCl_2_ and 0.2 g NaHCO_3_ in 1 litre of ultrapure water, sterilized with a 0.22 µm filter) using a glass Hamilton microlitre syringe (Hamilton, Reno, NV, USA). Injections were made into the right side of the tail, 5 mm from the end of the body towards the posterior tip of the tail. Tadpoles were then placed into individual 200 ml cups with aged water to recover. The tadpoles were re-anaesthetized at 24 and 48 h post-injection, and the injection sites were re-photographed. The degree of swelling in response to PHA was determined by subtracting the initial tail width from the tail width at 24 h or maximum 48 h post-injection from measurements made from images using the analysis program ImageJ. Likewise, a subset of metamorphs from the high- (*n* = 12) and low-UV-B groups (*n* = 8) were anaesthetized (Tricaine-S, 0.125 g l^−1^) and administered 5 µl of PHA (12 mg ml^−1^) using a BD Ultra-Fine II 0.3 ml 31 gauge insulin needle (North Ryde, NSW, Australia) subcutaneously into the skin surrounding the right triceps femoris. The thickness of the area where the injections were made was measured prior to the injections using fine-gauged digital callipers and again at 24 and 48 h post injection. Animals in which the swelling response was negative were excluded from further analysis. Final sample sizes for tadpole responses were as follows: UV-B treatment group, *n* = 8; and control group *n* = 6. Sample sizes for metamorph responses were as follows: UV-B treatment group, *n* = 6; and control group, *n* = 4.

### Leucocyte counts

Tadpoles (*n* = 19 and *n* = 15 for the UV-B treatment and control groups, respectively) and metamorphs (*n* = 11 and *n* = 8 for the UV-B treatment and control groups, respectively) were euthanized in buffered Tricaine-S (0.25 mg ml^−1^), and blood samples were obtained directly from the heart into heparinized capillary tubes. Blood smears were made, and leucocytes were stained with Quick Dipstain (POCD Healthcare, Artarmon, NSW, Australia). Blood smears were analysed at ×200 magnification (Olympus BH-2 microscope, Japan), and the percentage of leucocytes was determined for each animal from five different fields of view.

### Statistical analysis

All data were analysed using the statistical program R (R Core Team, 2013). Four tadpoles per tank within each treatment group were randomly selected and body size indices measured at three separate time points during the exposure period (at weeks 0, 2 and 4 after UV-B exposure began). The effects of UV-B on tadpole growth and developmental rates and tadpole immune function were analysed using a mixed-model approach, with ‘UV treatment’ as the fixed effect and ‘tank’ as a random factor. Tadpole metabolic rates were measured at two different time points during the experiment, once at 2 weeks after exposure began and again at 5 weeks. The data obtained from the first measurement at 2 weeks were analysed using a mixed-model approach, with ‘tank’ as a random factor and ‘body mass’ as a covariate. The data obtained from the second measurement at 5 weeks were analysed using a weight least-squares regression, using ‘tank’ as a random factor and ‘body mass’ as a covariate. The time taken to reach metamorphosis, mass at metamorphosis and length at metamorphosis were also analysed using mixed-effects models, with ‘UV treatment’ as the fixed factor and ‘tank’ as a random factor. The effect of UV-B treatment on the swelling response of metamorphs (one individual from each tadpole ‘tank’) following PHA injection was analysed using a robust regression method, and the effect of tadpole UV-B exposure on metamorph white blood cell counts was examined using an anlysis of variance (one metamorph from each tadpole ‘tank’).

## Results

### Growth rate and development

**Figure 1:**
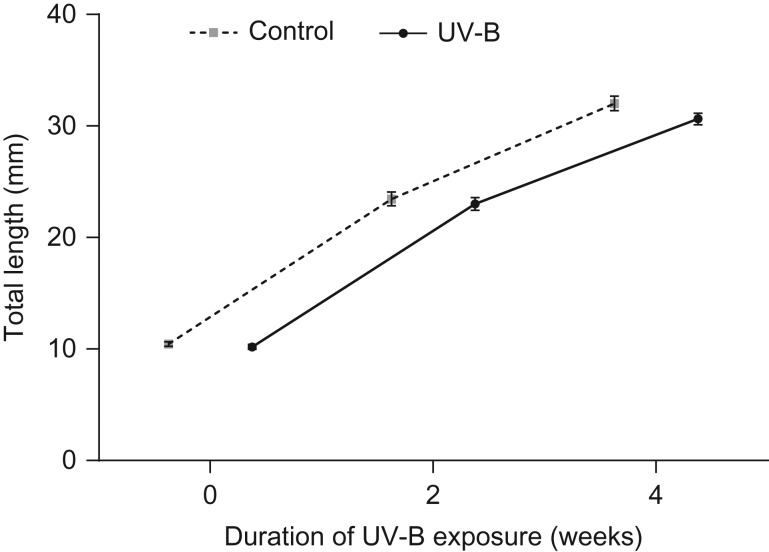
The mean length (in millimetres) of *Limnodynastes peronii* tadpoles in the ultraviolet-B (UV-B) treatment (*n* = 48) or control treatment (*n* = 36) measured at three different time points during development. There was no significant effect of larval UV-B exposure on growth rates (*F*_1,19_= 2.68, *P* = 0.12). Data are presented as mean values ± SEM.

There was no significant effect of larval UV-B exposure on the growth rate of tadpoles (*F*_1,19_ = 2.68, *P* = 0.12; Fig. 1). There was no effect of treatment on the developmental rate of tadpoles at week 4 of exposure (*F*_1,19_ = 0.62, *P* = 0.44), or their mass at metamorphosis (*F*_1,96_ = 0.44, *P* = 0.51; Table 3). Time taken to reach metamorphosis and length at metamorphosis were unaffected by UV-B exposure (*F*_1,19.7_ = 0.02, *P* = 0.89 and *F*_1,96_ = 2.98, *P* = 0.09, respectively; Table 3).

**Table 3:** The effect of 6 weeks of UV-B exposure during the tadpole stage on the time to metamorphosis and size at metamorphosis in *Limnodynastes peronii*

Metamorph traits	Control	UV-B
Time to metamorphosis (days)	77.79 ± 1.87	78.13 ± 1.58
Mass at metamorphosis (g)	0.3 ± 0.01	0.31 ± 0.01
Snout-to-vent length (mm)	15.7 ± 0.2	16.1 ± 0.2

Values are presented as means ± SEM. Abbreviation: UV, ultraviolet.

### Metabolic rate of tadpoles

**Figure 2:**
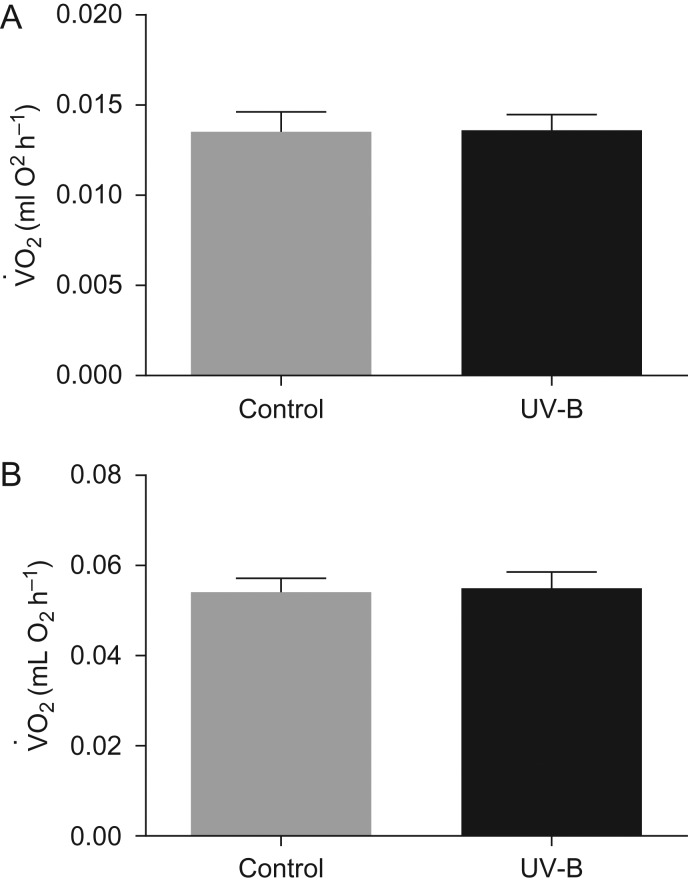
The standard metabolic rate (${\dot{V}}_{O_{2}}$; in millilitres of O_2_ per hour) of *L. peronii* tadpoles exposed to control and UV-B treatments measured during the initial stage of exposure (**A**; experienced from week 0 to 3 of the exposure period; absolute irradiance of UV-B during peak = 12.9 ± 3.18 µW cm^−2^; control, *n* = 28 and UV-B, *n* = 35) and during the final stage of exposure (**B**; experienced from week 3 to 6 of the exposure period; absolute irradiance of UV-B during peak = 18.41 ± 3.55 µW cm^−2^; control, *n* = 27 and UV-B, *n* = 25). There was no significant effect of larval UV-B exposure on standard metabolic rate rate at either the initial or the final stage of the exposure period. Data are presented as mean values + SEM.

Standard metabolic rates (${\dot{V}}_{O_{2}}$; in millilitres of O2 per hour) of tadpoles were significantly influenced by body mass at both 2 and 5 weeks of UV-B exposure (*F*_1,33_ = 48.2, *P* ≤ 0.001 and *F*_1,58.9_ = 42.27, *P* ≤ 0.001, respectively) but were unaffected by UV-B levels at both points (*F*_1,16_ = 0.21, *P* = 0.65 and *F*_1,19.1_ = 0.13, *P* = 0.72, respectively; Fig. 2).

### Immune function of tadpoles

**Figure 3:**
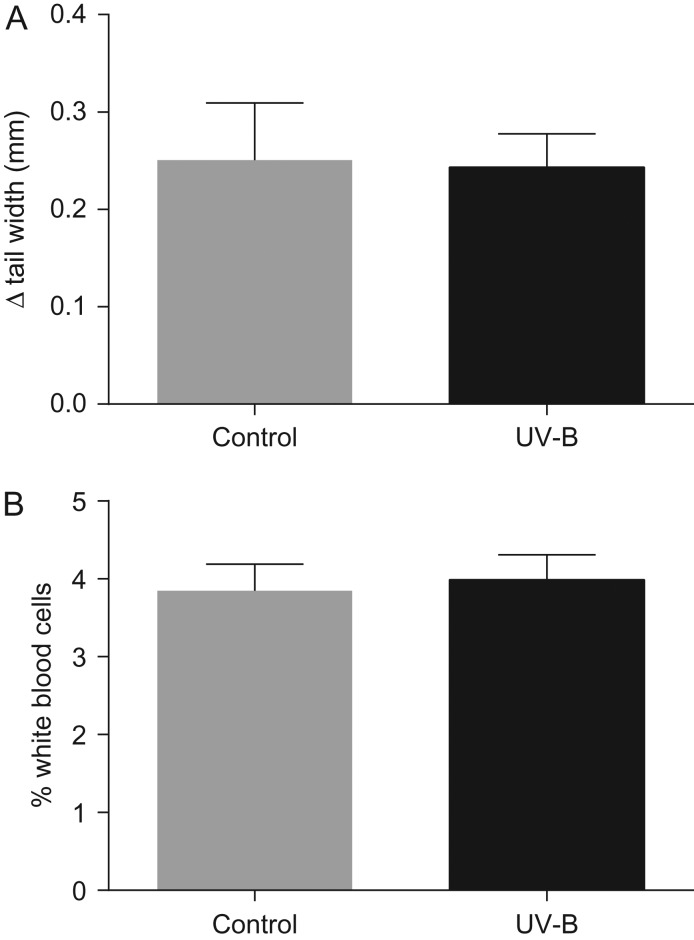
(**A**) The mean maximal tail swelling response (in millimetres) to phytohaemagglutinin administration in *L. peronii* tadpoles in the control treatment (*n* = 6) and UV-B treatment (*n* = 8). (**B**) The mean percentage of leucocytes per 200 blood cells in tadpoles exposed to the control treatment (*n* = 15) and UV-B treatment (*n* = 19). There was no significant effect of UV-B exposure on either parameter. Data are presented as mean values + SEM.

There was no significant effect of UV-B treatment on indices of tadpole immune function. Exposure to UV-B radiation did not affect the tissue swelling response to PHA injection (*F*_1,11_ = 0.2, *P* = 0.67; Fig. 3A) nor the proportion of leucocytes in the blood (*F*_1,18.2_ = 0.009, *P* = 0.92; Fig. 3B).

### Immune function of metamorphs

**Figure 4:**
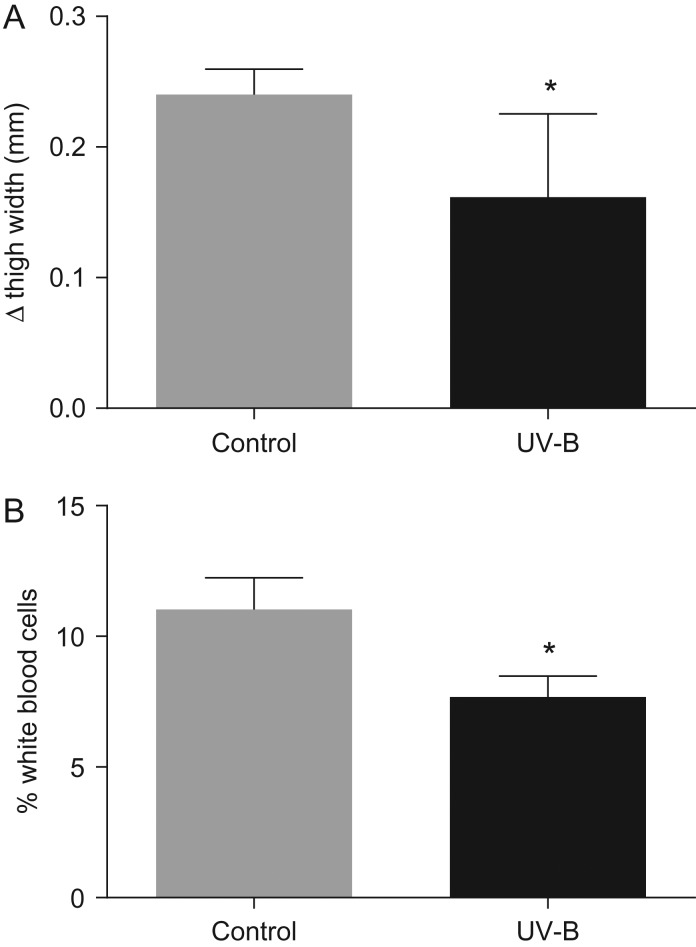
(**A**) The mean maximal thigh swelling response (in millimetres) to phytohaemagglutinin injection of *L. peronii* metamorphs in the control treatment (*n* = 4) and UV-B treatment (*n* = 6) during development as tadpoles. (**B**) The mean percentage of leucocytes of *L. peronii* metamorphs in the UV-B treatment (*n* = 11) and control treatment (*n* = 8) during development as tadpoles. The maximal response to phytohaemagglutinin was greatest in the metamorphs from the control treatment group (*F*_1,8_ = 0.93, *P* = 0.002). The proportion of leucocytes was also greatest in the control treatment group (*F*_1,17_ = 5.6, *P* = 0.03). *Significant difference (*P* < 0.05) between the UV-B and control treatment groups.

Early exposure to UV-B had a significant effect on the immune function of metamorphs. Metamorphs exposed to UV-B as tadpoles exhibited a 30% weaker response to PHA injection relative to the metamorphs not exposed to UV-B as tadpoles (*F*_1,8_ = 0.93, *P* = 0.002; Fig. 4A). The leucocyte count of metamorphs exposed to high UV-B as tadpoles was 4.1% lower in comparison to the metamorphs not exposed to UV-B as tadpoles (*F*_1,17_ = 5.6, *P* = 0.03; Fig. 4B).

## Discussion

We showed that UV-B exposure in early amphibian life stages had no immediate effect on the immune function of tadpoles, but elicited a carry-over effect on metamorphs, demonstrated by a decreased abundance of leucocytes and a reduced response to foreign antigen challenge. Despite the relatively low levels of UV-B received by tadpoles during the study, an effect on subsequent metamorph immune parameters was nonetheless apparent. Similar effects of UV-B exposure on fish and mammalian immune function have previously been established (Jeevan and Kripke, 1990; Jokinen *et al*., 2008), but this is the first time that an effect from prior UV-B exposure on immune function has been identified in amphibians.

This carry-over effect in metamorphs indicates that although UV-B exposure did not appear to have a direct effect on larval immune function, the development of the adult-type immune system was somehow disrupted by exposure to UV during the larval stages. The mechanism by which this disruption occurred remains unclear; however, it is possible that the cost of repair of UV-B-induced damage, although not significant enough to impair tadpoles, could have compromised the development of the immune system of individuals later in life. For example, tadpole growth or development rates that are faced with exposure to UV-B have an increased risk of predation, therefore impacting subsequent fitness (Alton *et al*., 2010). A similar trade-off may occur in immune function as a response to UV-B, in which tadpoles are able to manage the immediate damage caused by UV-B, but the development of resulting metamorphs is compromised. Mechanisms to repair UV-associated damage are inherent within cells (photolyase and nucleotide excision repair pathways), although activation of these processes incurs an energetic cost (Sancar and Tang, 1993). It is possible that the carry-over effect of early UV-B exposure is a product of a trade-off for resource allocation between immune function development and UV-B-induced repair mechanisms, leading to decreased immunocompetence in metamorphs. The effect of early larval UV-B exposure on subsequent immune function in metamorphs could also be due to direct damage to genes and/or the biological machinery responsible for the development of adult-type immune function. Whether the effects of UV-B on immune function exist as a result of direct damage or indirectly, as a result of an energy allocation trade-off, remains undetermined.

Whether the impact of early UV-B exposure on metamorph immune function remains an irreversible consequence of damage acquired during the larval period or reflects a more temporary response is unknown. During metamorphosis, amphibians experience a natural phase of immunosuppression, during which the immune system is reorganized from the ‘larval-type’ into the ‘adult-type’ immune system (Flajnik *et al*., 1987). In the period leading up to metamorphosis, the number of circulating leucocytes in the blood declines substantially until development of the ‘adult-type’ immune system commences, in which leucocyte density is again restored (Du Pasquier and Weiss, 1973). Metamorphs exposed to UV-B as tadpoles had lower leucocyte densities than those that received no UV-B as tadpoles, a potential indicator of an underdeveloped or immature immune system. A decrease in leucocytes in metamorphs suggests that exposure to UV-B may have disrupted white blood cell proliferation during development of the ‘adult-type’ immune system, resulting in fewer cells at the completion of metamorphosis. It is possible that larval exposure to UV-B might have delayed development of the adult-type immune system and that, given time, full immunocompetence could be restored. Alternatively, exposure to UV-B during development might have resulted in permanent damage to immune function, so that the immune function remains in an underdeveloped state. Analysis of immunocompetence at additional time points following metamorphosis is required to determine whether immune function is delayed or permanently disrupted following larval UV-B exposure.

The results of this study have important implications for our understanding of disease risks in amphibians. Our data show that early larval UV-B exposure can influence the subsequent immune capacity of resulting juvenile frogs, which has the potential to increase their risk of susceptibility to pathogens such as *Bd*. Moreover, amphibian species differ in their susceptibility to pathogens such as *Bd* (e.g. Briggs *et al*., 2010) and also in their capacity to repair UV-B-induced DNA damage (van de Mortel *et al*., 1998). However, whether UV-B exposure increases the susceptibility of amphibian larvae to *Bd* infection remains unclear. Several studies have shown contrasting results; simultaneous UV-B and *Bd* exposure either has no effect on infection risk in amphibian larvae (Garcia *et al*., 2006; Searle *et al*., 2010) or the two factors interact antagonistically to lower infection risk (Ortiz-Santaliestra *et al*., 2011). However, these studies do not take into consideration the potential for early UV-B exposure to have a latent influence on *Bd* susceptibility in subsequent developmental stages. The results of the present study suggest that the impacts of UV-B may be long lasting, and further work in this area is needed to elucidate the mechanisms through which UV-B may influence disease susceptibility through immune system dysfunction.

The risk to effective amphibian conservation lies in the inability of immunocompromised individuals to respond adequately to pathogens, which may elevate their risk of disease (Berger *et al*., 1998; Longcore *et al*., 1999; Voyles *et al*., 2007, 2009; Garner *et al*., 2009). Therefore, decreased immune function in metamorphs, as a carry-over effect of UV-B exposure, has the potential to increase susceptibility to *Bd* and chytridiomycosis, given that amphibians are most vulnerable to this pathogen during the post-metamorphosis life stage (Berger *et al*., 1998; Pessier *et al*., 1999). Other studies have shown that physiological stressors applied in the larval period of amphibians can influence immunological fitness in the resulting metamorphs (Gervasi and Foufopoulos, 2008). Like UV-B exposure, the impacts of early life-history stresses may not necessarily manifest immediately, but may subsequently influence immune function and disease susceptibility following metamorphosis. Predictions of the effects of early larval exposure to environmental stressors such as UV-B on disease ecology should not be restricted to *Bd*; immunosuppression and/or delayed immune system development in post-metamorphic amphibians could be detrimental in the presence of any other disease-causing pathogens.

In light of recent dramatic amphibian declines, the results of the present study provide important insight for our understanding of UV-B radiation as an influential stressor of amphibians. This study demonstrates that exposure to environmental stressors, such as UV-B radiation, during early development could impose significant fitness consequences for individuals later in life as a result of the impact of UV-B on immune function.
